# High-dose glucocorticoids in COVID-19 patients with acute encephalopathy: clinical and imaging findings in a retrospective cohort study

**DOI:** 10.1007/s00702-024-02751-9

**Published:** 2024-02-16

**Authors:** Alexandra Rhally, Giulia Bommarito, Marjolaine Uginet, Gautier Breville, Patrick Stancu, Alice Accorroni, Frédéric Assal, Patrice H. Lalive, Karl-Olof Lövblad, Gilles Allali

**Affiliations:** 1 Faculty of Medicine, University of Geneva, Geneva, Switzerland; 2 Department of Clinical Neurosciences, Lausanne University Hospital and University of Lausanne, Lausanne, Switzerland; 3 Division of Neurology, Department of Clinical Neurosciences, Faculty of Medicine, Geneva University Hospitals, University of Geneva, Geneva, Switzerland; 4 Division of Laboratory Medicine, Diagnostic Department, Geneva University Hospitals, Geneva, Switzerland; 5 Department of Pathology and Immunology, Faculty of Medicine, University of Geneva, Geneva, Switzerland; 6 Division of Neuroradiology, Geneva University Hospitals and University of Geneva, Geneva, Switzerland; 7 Division of Cognitive and Motor Aging, Department of Neurology, Albert Einstein College of Medicine, Yeshiva University, Bronx, NY USA; 8 Leenaards Memory Center, Lausanne University Hospital and University of Lausanne, Lausanne, Switzerland

**Keywords:** COVID-19, Acute encephalopathy, High-dose glucocorticoids (GC), MRI, Microbleeds, White matter hyperintensities

## Abstract

**Objectives:**

Acute encephalopathy (AE) has been described as a severe complication of COVID-19. Inflammation has been suggested as a pathogenic mechanism, with high-dose glucocorticoids (GC) showing a beneficial effect. Here, we retrospectively analyzed the clinical and radiological features in a group of COVID-19 AE patients who received GC treatment (GT) and in a non-treated (NT) group.

**Method:**

Thirty-six patients with COVID-19 AE (mean age 72.6 $\pm$ 11 years; 86.11% men) were evaluated for GC treatment. Twelve patients (mean age 73.6 $\pm$ 4.5 years; 66.67% men) received GC, whereas 24 patients who showed signs of spontaneous remission were not treated with GC (mean age 70.1 $\pm$ 8.6 years; 95.83% men). Differences in clinical characteristics and correlations with imaging features were explored.

**Results:**

The GT group showed signs of vulnerability, with a longer hospitalization (*p* = 0.009) and AE duration (*p* = 0.012) and a higher hypertensive arteriopathy (HTNA) score (*p* = 0.022), when compared to NT group. At hospital discharge, the two groups were comparable in terms of clinical outcome (modified Rankin scale; *p* = 0.666) or mortality (*p* = 0.607). In our whole group analyses, AE severity was positively correlated with periventricular white matter hyperintensities (*p* = 0.011), deep enlarged perivascular spaces (*p* = 0.039) and HTNA score (*p* = 0.014).

**Conclusion:**

This study suggests that, despite signs of radiological vulnerability and AE severity, patients treated by high-dose GC showed similar outcome at discharge, with respect to NT patients. Imaging features of cerebral small vessel disease correlated with AE severity, supporting the hypothesis that brain structural vulnerability can impact AE in COVID-19.

## Introduction

During the pandemic, acute encephalopathy (AE), in its most frequent clinical manifestation, delirium, affected up to 65% of hospitalized patients with COVID-19 (Helms et al. 2020) and was associated with a higher mortality (Mendes et al. 2021). The risk of developing delirium in COVID-19 was greater in older patients, in presence of a more severe form of the disease, and of a preexisting cognitive impairment (Wilke et al. 2022; Damanti et al. 2023). Several factors likely contribute to AE pathogenesis in COVID-19, with suggested mechanisms including exposure to intensive care unit procedures, a prothrombotic, microvascular dysfunction, and proinflammatory state (Pensato et al. 2021). Indeed, inflammation at both peripheral and central level has been associated with delirium (Bernard-Valnet et al. 2021; Fajgenbaum and June 2020) and a case series observed in the Geneva University Hospitals reported a good response to high-dose intravenous GC (Pugin et al. 2020). Based on these findings, in 2020, the interdisciplinary medical board of the Geneva University Hospitals approved an experimental protocol to identify COVID-19 patients with AE and, under specific conditions, treat them with high-doses of GC.

Here, we report the results of a retrospective clinical study, by describing clinical features of the cohort of COVID-19 AE patients who were included in a protocol evaluating the eligibility to a high-dose GC treatment. Within this group, we assessed differences in clinical and imaging features between patients who were treated by GC and those who did not receive treatment. We also evaluated whether imaging features indicative of inflammation (endotheliitis) or microangiopathy [cerebral small vessel disease (CSVD)] were associated with AE severity and clinical outcome at discharge.

## Materials and methods

### Participants

Ninety-eight patients hospitalized in the Geneva University Hospitals from April 2020 to May 2021, were diagnosed with SARS-CoV-2 infection and presented signs of AE. SARS-CoV-2 infection was confirmed by a positive SARS-CoV-2 reverse transcription polymerase chain reaction assay from a nasopharyngeal swab at the time of hospitalization. Out of those patients, 36 patients met the criteria of severe AE and were evaluated for GC administration.

The decision to treat with high-dose GC was based on an algorithm conceived by a multidisciplinary expert panel and approved by the institutional review board of the Geneva University Hospitals. Specifically, the criteria to treat with GC included (i) a Richmond agitation sedation scale (Sessler et al. 2002) (RASS) < -3 or the combination of an RASS ≥ -3 and (1) a confusion assessment method (Inouye et al. 1990) (CAM) ≥ 3 or (2) mutism, (ii) the exclusion of brain lesion, status epilepticus, other common causes of delirium (hypoxic, metabolic, toxic, etc.), and encephalitis that could explain the neurological symptoms, (iii) the presence of gadolinium enhancement at the level of intracranial arterial walls (assessed by three board-certified neuroradiologists and validated when common agreements were reached) (Fig. 2E and F), and (iv) the absence of signs of spontaneous remission within 48 h of protocol inclusion.

**Fig. 1:**
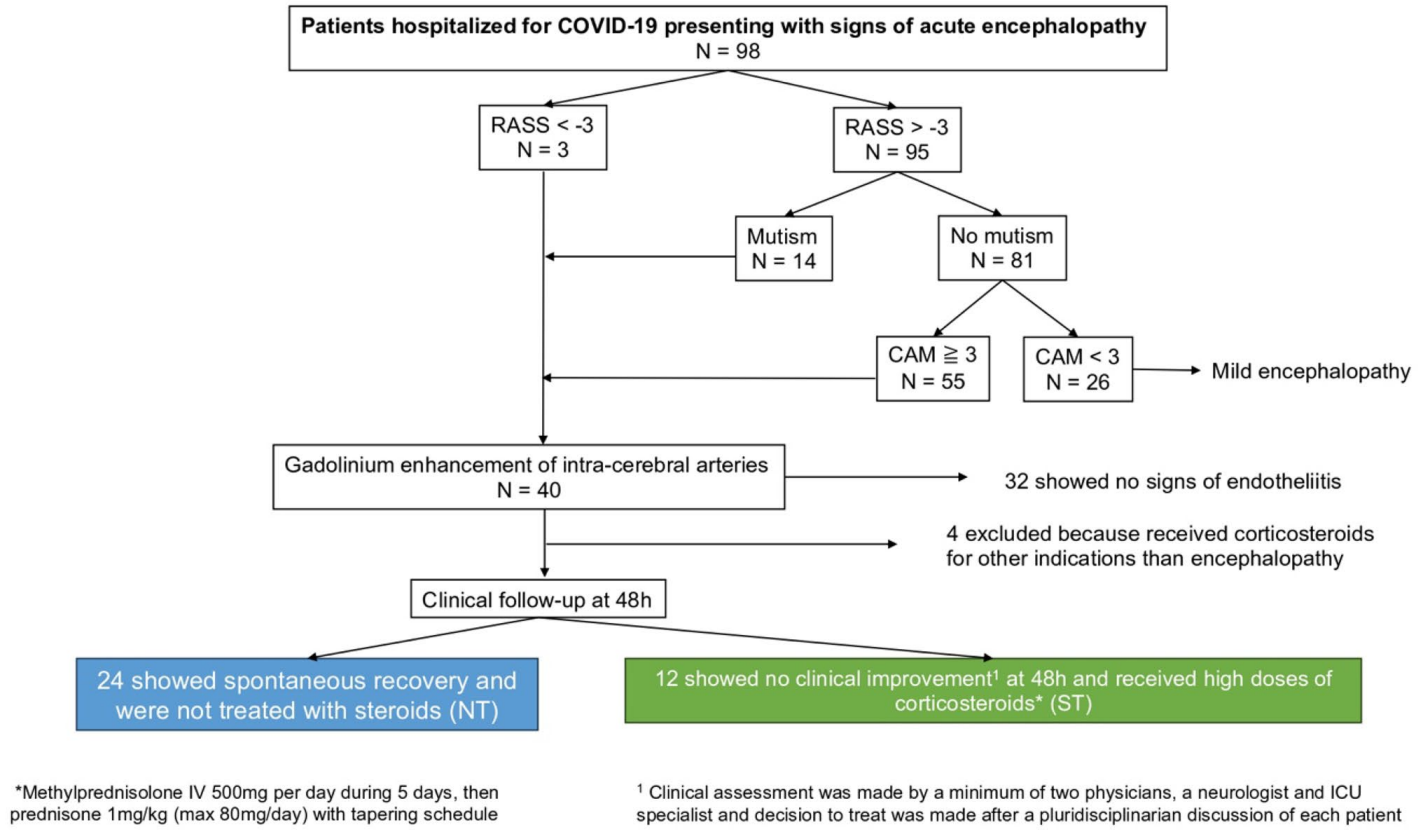
Flowchart of inclusion/exclusion criteria for GC, for hospitalized COVID-19 patients presenting with AE

After careful consideration, 12 patients, who met all four criteria, were treated with high-dose GC (GT group). Treatment protocol consisted of the intravenous administration of 500 mg of methylprednisolone per day for 5 days, followed by prednisone 1 mg/kg (max. 80 mg/day) with tapering schedule. The non-treated (NT) group included 24 patients who met all first 3 criteria but who did not receive GC because of spontaneous improvement within the 48 h after the first neurological evaluation (Fig. 1). Clinical data was retrospectively retrieved from patients’ charts.

### Standard protocol approvals, registrations, and patients consents

The study was approved by the institutional review board of Geneva University Hospitals (protocol #2020-01206–approved May 25, 2020) and has been performed in accordance with the Declaration of Helsinki.

### Brain imaging

All patients in the GT and NT groups underwent an MRI on a Philips Ingenia (Philips Medical Systems, Eindhoven, The Netherlands) 1.5 T scanner equipped with a head and neck coil at the Geneva University Hospitals, within on average 6 days from AE symptoms onset. Time lag between the neurological symptoms onset and MRI acquisition was comparable between groups (mean GT = 7.18 $\pm$ 6.3 days, mean CG = 5.5 $\pm$ 4.1 days, *p* = 0.349).

Sequences included a T2-weighted (repetition time (TR): 3600 ms, echo time (TE): 100 ms, slice thickness 4 mm), susceptibility-weighted images (SWI, TR: 520 ms, TE: 0 ms, slice thickness 2 mm), a fluid-attenuated inversion recovery imaging (FLAIR, TR: 9000 ms, TE: 120 ms, slice thickness 4 mm), and a dynamic 3D contrast-enhanced MRA (TE: 17 ms, TR: 400 ms, image thickness 1.5 mm) of the neck vessels was performed from the aortic arch to the circle of Willis. Pre-contrast and post-contrast fat-saturated T1-weighted black blood VISTA images in all patients were acquired in the axial and coronal planes.

**Fig. 2:**
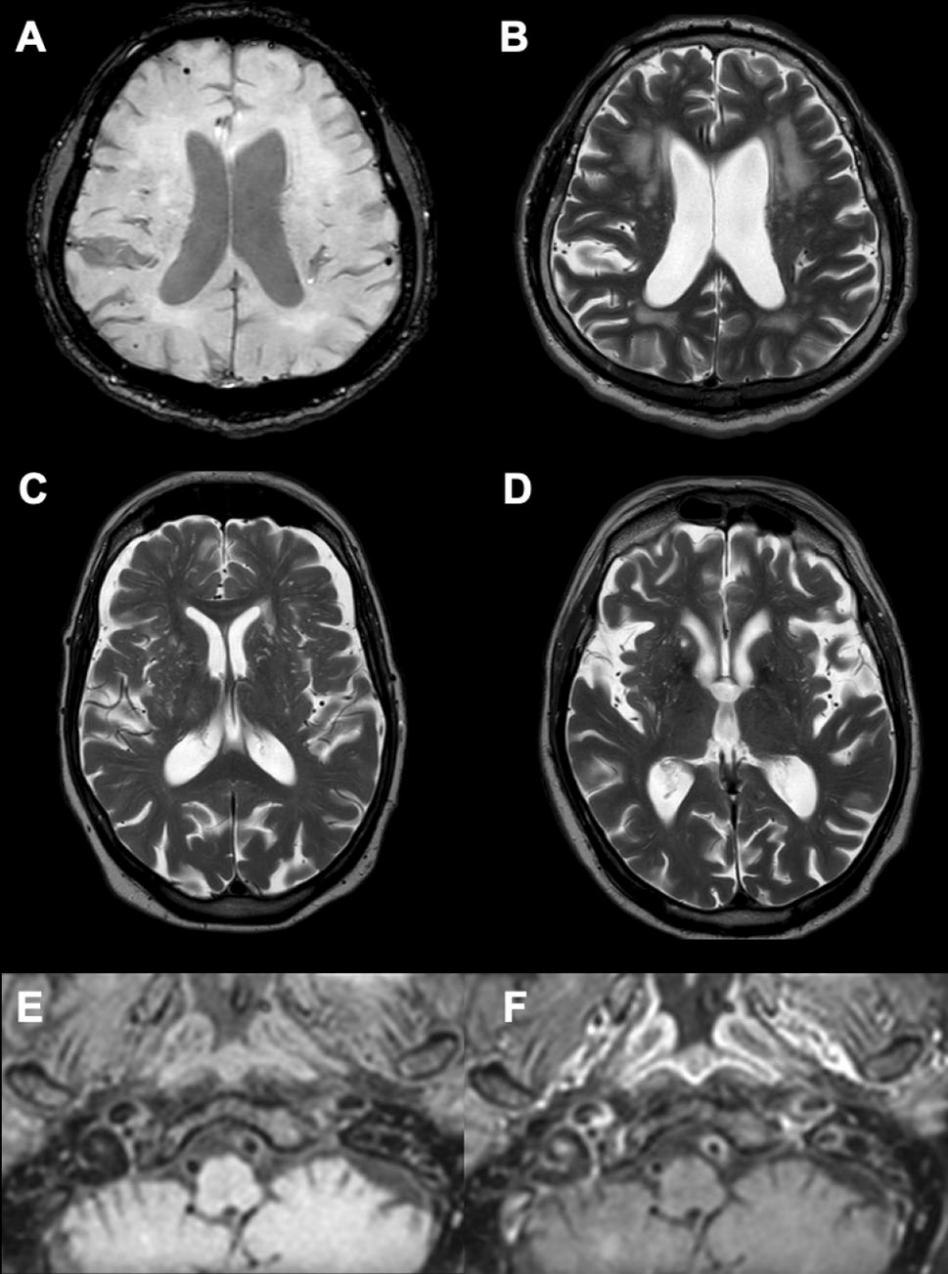
Imaging features explored: microbleeds on SWI (**A**), white matter hyperintensities (**B**), EPVS (**C**), and lacunes (**D**) on T2-weighted, and endotheliitis as suggested by enhancement of the vessel wall of the left vertebral artery on VISTA sequences after gadolinium injection (**F** vs pre-contrast **E**)

The following imaging features were considered: the number of vessels presenting walls enhancement, microbleeds, white matter hyperintensities (WMH), lacunes, cortical superficial siderosis, and enlarged perivascular spaces (EPVS) (Fig. 2).

Contrast enhancement of the vessel walls was retained as a possible marker of inflammation when homogeneous and circumferential (greater than 50% of the circumference) (Uginet et al. 2022a). Inflammatory atheromatous plaques, as a potential cause of such intracranial vessel enhancement, were excluded by angio‐MR, angio‐CT, or echo‐Doppler. Enhancement detection and number of vessels involved were ascertained by two board-certified neuroradiologists and validated when common agreements were reached.

The severity of white matter hyperintensities was assessed using the Fazekas scale (Fazekas et al. 2002).

The microbleeds anatomical rating scale (MARS) (Gregoire et al. 2009) was used to quantify the number and location of microbleeds, which were subsequently grouped into lobar, deep, and infratentorial.

The burden of EPVS was assessed on axial T2-weighted images in the basal ganglia and centrum semi-ovale and stratified into: < 10 EPVS, 11–20 EPVS, and > 20 EPVS. Lacunes were defined as rounded or ovoid lesions, with a diameter between 3 and 20 mm, with CSF signal density on T2-weighted images.

Rating was performed independently by two investigators (AR and GB), under the supervision of the head of the Neuroradiology Unit (KOL). In the case of discrepant ratings, a consensus was accomplished subsequently, by a third person (GA). As microbleeds have been associated to critical illness, we compared microbleeds rate between patients admitted or not to ICU.

The CSVD, cerebral amyloid angiopathy (CAA), and hypertensive arteriopathy (HTNA) scores was computed according to a previous study (Lau et al. 2017). Specifically, for CSVD score, one point was given for presence of (i) lacunes, (ii) 1–4 microbleeds, (iii) moderate-to-severe BG EPVS (> 20), and (iv) moderate WMH (total periventricular + deep WMH score 3–4), while 2 points were given for the presence of (i) ≥ 5 CMBs, (ii) severe WMH (total periventricular + deep WMH score 5–6). For CAA score, one point was given for the presence of (i) 1–4 lobar microbleeds, (ii) moderate-to-severe semi-oval center EPVS (> 20), (iii) deep WMH ≥ 2 or periventricular WMH > 3, and (iv) focal cSS, and two points for (i) ≥ 5 lobar microbleeds and (ii) disseminated cSS. For HTNA score, one point was allocated for (i) lacunes, (ii) periventricular WMH > 3 or deep WMH 2–3, (iii) ≥ 1 deep microbleeds, and (iv) presence of moderate-to-severe basal ganglia EPVS (> 10 EPVS).

### Statistical analyses

The non-parametric Mann–Whitney *U* test was used to compare clinical and imaging features between groups. Spearman correlation test was used to correlate clinical and imaging features in the whole group. Statistical analysis was performed using Matlab (R2023a, Natick, Massachusetts: The MathWorks Inc).

## Results

### Clinical and imaging features in the two groups

Demographic and clinical data for the whole group of candidate patients to high-dose GC and for GT and NT groups are reported in Table 1. Patients were mainly men (66.67% and 95.83% in the GT and NT, respectively), with a median age of 74.1 $\pm$ 7.3 and 70.6 $\pm$ 12 years in the GT and NT, respectively. The GT and NT groups did not differ in terms of cardiovascular risk factors or preexisting cognitive disorder. When considering clinical features, rate of ICU admission, mortality, mRS at discharge, and median CAM and RASS scores were similar between groups. AE duration and hospitalization duration were longer in the GT compared to the NT (*p* = 0.012 and *p* = 009 respectively).

**Table 1:** Clinical data in the whole cohort, GT and NT

	Whole group (*n* = 36)	GT group (*n* = 12)	NT group (*n* = 24)	*P* value
Age (years)	72.6 (11)	74.1 (7.3)	70.6 (12)	*0.322*
Male sex, (%)	31 (86.11%)	8 (66.67%)	23 (95.83%)	***0.017***^e^
Cardiovascular risk factors				
BMI^a^ (kg/m^2^) (%)	27.3 (5.6)	28.2 (5.4%)	26.8 (5.8%)	*0.58*
Smoking, *N* (%)	8 (22.22%)	1 (8.33%)	7 (29.17%)	*0.156*^e^
Hypertension, *N* (%)	26 (72.22%)	9 (75%)	17 (70.83%)	*0.792*^e^
Diabetes, *N* (%)	16 (44.44%)	3 (25%)	13 (54.17%)	*0.097*^e^
Previous cognitive disorders, *N* (%)	3 (8.33%)	1 (8.33%)	2 (8.33%)	*1.00*^e^
Hospitalization duration (days)	32.5 (22)	46.0 (18.0)	29.0 (14.0)	***0.009***
Intensive care unit admission, *N* (%)	23 (63.89%)	8 (66.67%)	15 (62.5%)	*0.806*^e^
Mortality, *N* (%)	2 (5.56%)	1 (8.33%)	1 (4.17%)	*0.607*^e^
Acute Encephalopathy features				
AE duration (days)	12 (11)	18.5 (16.0)	11.0 (7.0)	***0.012***
RASS at first neurological consult^b^	− 1.0 (2.5)	− 2.0 (3.0)	0.0 (1.5)	*0.174*
CAM at first neurological consult^c^	3.0 (1.0)	3.5 (1.0)	3.0 (1.0)	*0.745*
mRS^d^ at discharge	3.0 (2.0)	3.0 (2.0)	3.0 (2.5)	*0.666*
Time lag between onset of AE and steroids’ treatment (days)	–	10.0 (8.5)	–	
Time lag between onset of steroids treatment and AE improvement (days)	–	5.0 (8.5)	–	

bold italic is are p-values that are significant, italic are p-values

^a^ Body Mass Index, ^b^ Richmond Agitation-Sedation Scale, ^c^ Confusion Assessment Method, ^d^ modified Rankin Scale, ^e^ Chi-square used. All values are reported as median (± IQR) if not otherwise specified

Regarding imaging features (Table 2), GT and NT showed a similar WMH load, microbleeds, lacunes, EPVS, cSS, CSVD score, CAA score, and number of vessels presenting signs of endotheliitis. However, the HTNA score was significantly different between groups, with a median score of 2 for the GT and 1 for the NT (*p* = 0.022).

**Table 2:** Imaging features in the whole cohort, GT and NT

	Whole group (*n* = 36)	GT group (*n* = 12)	NT group (*n* = 24)	*P* value
Endotheliitis, *N* of patients (%)	36 (100%)	12 (100%)	24 (100%)	*1.00*^a^
N of enhancing vessels per patient	2.0 (2.0)	2.0 (2.0)	2.0 (2.0)	*1.00*
White matter hyperintensities				
Periventricular (Fazekas)	1.0 (1.0)	1.0 (1.0)	1.0 (0.5)	*0.388*
Deep (Fazekas)	1.0 (1.0)	2.0 (1.0)	1.0 (1.5)	*0.142*
Microbleeds				
Total	1.0 (3.0)	1.0 (7.0)	1.0 (3.0)	*0*.*549*
Lobar	0.5 (3.0)	0.5 (5.0)	0.5 (3.0)	*0.652*
Deep	0.0 (1.0)	0.5 (1.0)	0.0 (0.0)	*0.098*
Infratentorial	0.0 (0.0)	0.0 (0.0)	0.0 (0.0)	*1.00*
Lacunes				
Count	0.0 (0.5)	0.0 (1.0)	0.0 (0.0)	*0.439*
EPVS				
Deep	10.5 (16.0)	12.0 (15.0)	9.0 (12.0)	*0.439*
Cortical	8.0 (18.0)	5.5 (30.0)	8.0 (14.0)	*0.8*
cSS	0.0 (0.0)	0.0 (0.0)	0.0 (0.0)	*0.175*
CSVD score	2.0 (1.5)	2.0 (1.0)	1.0 (2.5)	*0.057*
CAA score	1.0 (2.0)	1.5 (2.0)	1.0 (2.0)	*0.286*
HTNA score	1.0 (1.0)	2.0 (1.5)	1.0 (2.0)	***0.022***

bold italic is are p-values that are significant, italic are p-values

No significant differences in microbleeds rate were found between patients admitted or not to the ICU (*p* = 0.511).

We also noted a median of 10.0 $\pm$ 8.5 days between AE onset and GC treatment with a median recovery of 5.0 $\pm$ 8.5 days for the GT.

### Correlation analysis

Within our entire cohort (Table 3), the CAM score was positively correlated to periventricular WMH (*r* = 0.42, *p* = 0.011), to deep EPVS (*r* = 35, *p* = 0.039) and to the HTNA score (*r* = 0.41, *p* = 014). No other significant correlations were found between mRS and imaging features.

**Table 3:** Correlations between mRS, CAM and imaging features in our cohort

	CAM	mRS
N of enhancing vessels	*r* = 0.13, *p* = 0*.436*	*r* = 0.07*, p* = *0.696*
WMH		
Periventricular	***r* = 0.42, *p* = *0.011***	*r* = − 0.05,* p* = *0.762*
Deep	*r* = 0.24, *p* = *0.153*	*r* = − 0.10, *p* = *0.579*
Microbleeds		
Total	*r* = 0.03, *p* = *0.841*	*r* = − 0.16, *p* = *0.343*
Lobar	*r* = 0.02*. p* = *0.901*	*r* = − 0.17*, p* = *0.328*
Deep	*r* = 0.11, *p* = *0.533*	*r* = − 0.03, *p* = *0.852*
Infratentorial	*r* = − 0.06, *p* = *0.724*	*r* = − 0.03, *p* = *0.882*
Lacunes	*r* = 0.11, *p* = *0.531*	*r* = − 0.07, *p* = *0.671*
EPVS		
Deep	***r* = 0.35, *p* = *0.039***	*r* = 0.05, *p* = *0.753*
Cortical	*r* = − 0.07, *p* = *0.703*	*r* = − 0.01, *p* = *0.973*
cSS	*r* = 0.18, *p* = *0.304*	*r* = 0.18, *p* = *0.282*
CSVD score	*r* = 0.28, *p* = *0.094*	*r* = − 0.06, *p* = *0.717*
CAA score	*r* = 0.16, *p* = *0.365*	*r* = − 0.12, *p* = *0.47*
HTNA score	***r* = 0.41, *p* = *0.014***	*r* = 0.09, *p* = *0.586*

bold italic is are p-values that are significant, italic are p-values

## Discussion

This study was performed during the acute phase of the pandemic, as a follow-up to a previous study done in our center (Pugin et al. 2020). We report here the clinical and imaging features of COVID-19 patients with AE who were eligible to a treatment with high-dose intravenous GC, and compare clinical and imaging features with a non-treated group with spontaneous signs of remission. Our findings reveal that patients who were treated with high-dose GC had a similar disability at discharge, when compared to NT, despite signs of clinical and radiological vulnerability, as the lack of spontaneous improvement, longer hospitalization, longer AE duration, and higher HTNA score. Moreover, delirium severity was related to radiological features of microvascular damage but not of inflammation.

High-dose GC have been used to successfully treat patients with COVID-19 AE, with an improvement in the 48–72 h after administration (Pugin et al. 2020, Pizzato Tondo et al. 2021), and other neurological manifestations COVID-19 related (Pilotto et al. 2020). Indeed, an inflammatory process, involving the central nervous system in a direct or indirect way, through a cytokine storm, has been implied in the pathogenesis of neurological manifestations in COVID-19, including delirium. This has been corroborated by radiological and pathological findings showing signs of endotheliitis (Uginet et al. 2022b, Varga et al. 2020) and inflammatory syndromes involving the central nervous system (Uginet et al. 2022b, Paterson et al. 2021).

Based on these premises, following the case series (Pugin et al. 2020) done at our hospital and as a last resort given the lack of signs of clinical improvement, we decided to treat COVID-19 patients with AE with high-dose GC when eligible, based on specific criteria (Fig. 1). Our findings show that, though showing several signs of severity and lack of spontaneous remission, the GT rapidly improved after high-dose GC administration and showed similar functional outcome at discharge when compared to less severe AE patients not requiring high-dose GC treatment. Moreover, mortality rate was similar between groups. Both these observations lead us to suggest that GC treatment was justified in those patients and support inflammation as an etiological hypothesis in COVID-19 AE. This finding is corroborated by other more recent studies, showing that the use of high-dose GC in conditions associated with delirium can help to prevent it (Awada et al. 2022; Xiang et al. 2022) or to reduce its severity (Kluger et al. 2021). Further studies to properly define high-dose GC in preventing or treating delirium in populations at risk are needed.

In terms of brain imaging, we assessed whether imaging features of inflammation or microangiopathy were correlated to clinical severity of COVID-19 AE. The number of enhanced vessels was not related to the severity or clinical outcome of our patients, confirming our previous observation (Uginet et al. 2021). Possibly, vessels enhancement does not completely capture the inflammation process associated with COVID-19 AE, and more advanced imaging features reflecting parenchymal inflammation could better explain severity and predict outcome in COVID-19 AE.

Imaging features suggestive of CSVD, as microbleeds, WMH, and lacunes, have been described in COVID-19 patients and they have been associated with an increased mortality, critical illness, and worse functional outcome (Agarwal et al. 2020). The high rate of microbleeds observed in patients with COVID-19 could be a direct consequence of critical illness (Kirschenbaum et al. 2021). However, we found that the ICU admission rate was not different between the two groups and that patients admitted to ICU did not present with a higher rate of microbleeds. Thus, microbleeds together with WMH, lacunes, and EPVS could represent markers of preexisting CSVD. Indeed, mechanisms other than inflammation are likely to be involved in the severity of COVID-19 AE and brain endothelial damage has been widely described in patients with COVID-19. In our cohort, patients with a higher HTNA score had a more severe form of encephalopathy, independently of GC treatment. Thus, we can speculate that a preexisting frailty at a microvascular level can predispose to AE severity. The quantification of microvascular damage by use of imaging could shed light into the pathogenesis of COVID-19 AE, especially as the link between endothelial damage and neurovascular brain changes seen in hypertensive arteriopathy has already been linked to delirium in the postoperative phase, as well as cognitive decline in previous studies (Kant et al. 2017). In our entire cohort, AE clinical severity in the acute phase was also associated with a higher WMH load, especially in the periventricular regions, as well as deep EPVS. Presence of white matter hyperintensities, as well as EPVS, is a marker of CSVD and is associated with neurodegeneration and cognitive impairment (Matsuda et al. 2021). Therefore, these findings support the hypothesis that patients with imaging markers of an underlying neurodegenerative process were more prone to develop a more severe form of COVID-19 AE.

This raises the questions of an underlying vulnerability for severe AE in patients with recognized or unrecognized neurodegenerative conditions, but also for possible long-term effects of COVID-19 infection (Bommarito et al. 2023). Systemic inflammation, acute respiratory distress syndrome, and sepsis are known factors to contribute to long-term development of neurological consequences and cognitive decline, either accelerating an ongoing neurodegenerative process or being the onset of a new pathological process (Iwashyna et al. 2010). Future studies should investigate the correlation between COVID-19 AE and possible higher prevalence of cognitive decline in COVID-19 AE survivors (Heneka et al. 2020).

Although this sample of COVID-19 patients with brain imaging during the acute stage of their AE, as well as the thorough adherence to protocol regarding the selection of patients for treatment are a strength of this study, we should acknowledge some limitations. First, the small sample size of our cohort, due to an insufficient number of patients presenting with COVID-19 AE and responding to the inclusion criteria, limits the power of the study and thus may limit the generalization of the study’s findings. Second, the retrospective design of the study brings some limitations, including the incomplete inclusion of confounding factors that could have influenced AE severity, quantitative MRI data, and the absence of optimal control group(s). Specifically, as the data refer to the acute phase of the pandemic, first and second wave, we miss clinical and laboratory data, as a comprehensive list of precipitating factors for AE other than COVID-19, that could have helped to better explain AE duration and outcome. Moreover, we lack a control group of COVID-19 patients without AE which would enable us to specify if the white matter hyperintensities and enlarged perivascular spaces patterns described here are pathogenic to COVID-19 AE or not. We also lack a control group of AE patients without COVID-19, so we cannot conclude that our findings are specific to COVID-19 infection. Finally, the decision to reevaluate patients at 48 h specifically after the first neurological consult was based on experts’ opinions and “clinical improvement at 48 h” was a qualitative appreciation based on the clinical expertise of two physicians (one neurologist and one critical care physician), which could lead to biases because of the lack of quantitative criteria for improvement. A randomized-controlled trial, with a larger sample size, would allow us to confirm these preliminary observations.

## Conclusions

This study suggests that severe COVID-19 AE patients treated with high-dose GC showed a favorable clinical outcome at discharge, similar to patients with spontaneous improvement, suggesting high-dose GC benefit as an acute treatment in some COVID-19 AE patients. White matter changes as well as other signs of CSVD both contribute to severity and clinical outcome of AE in COVID-19. Such findings can be useful in identifying patients at high risk of developing AE in COVID-19 or other predisposing conditions, and to refine treatment protocols.

## Data Availability

De-identified data will be made available to qualified investigators upon written request to the last author.
